# A novel method of identifying inner ear malformation types by pattern recognition in the mid modiolar section

**DOI:** 10.1038/s41598-021-00330-6

**Published:** 2021-10-21

**Authors:** Anandhan Dhanasingh, Daniel Erpenbeck, Masoud Zoka Assadi, Úna Doyle, Peter Roland, Abdulrahman Hagr, Vincent Van Rompaey, Paul Van de Heyning

**Affiliations:** 1 Research and Development Department, MED-EL Medical Electronics, Fürstenweg77a, 6020 Innsbruck, Austria; 2 Department of Translational Neurosciences, Faculty of Medicine and Health Sciences, University of Antwerp, Antwerp, Belgium; 3 Department of Otorhinolaryngology and Head and Neck Surgery, Antwerp University Hospital, Antwerp, Belgium; 4 ARTORG Center for Biomedical Engineering Research, University of Bern, Bern, Switzerland; 5 Department of Otolaryngology, University of Texas Southwestern Medical Center, Dallas, USA; 6 King Abdullah Ear Specialist Center (KAESC), King Saud University, Riyadh, Saudi Arabia

**Keywords:** Outcomes research, Translational research, Neuroscience, Anatomy, Neurology, Signs and symptoms

## Abstract

Identification of the inner ear malformation types from radiographs is a complex process. We hypothesize that each inner ear anatomical type has a uniqueness in its appearance in radiographs. The outer contour of the inner ear was captured from the mid-modiolar section, perpendicular to the oblique-coronal plane, from which the A-value was determined from CT scans with different inner ear anatomical types. The mean A-value of normal anatomy (NA) and enlarged vestibular aqueduct syndrome (EVAS) anatomical types was greater than for Incomplete Partition (IP) type I, II, III and cochlear hypoplasia. The outer contour of the cochlear portion within the mid-modiolar section of NA and EVAS resembles the side view of Aladdin’s lamp; IP type I resembles the side-view of the Sphinx pyramid and type II a Pomeranian dog’s face. The steep spiraling cochlear turns of IP type III resemble an Auger screw tip. Drawing a line parallel to the posterior margin of internal auditory canal (IAC) in axial-view, bisecting the cavity into cochlear and vestibular portions, identifies common-cavity; whereas a cavity that falls under the straight-line leaving no cochlear portion identifies cochlear aplasia. An atlas of the outer contour of seventy-eight inner ears was created for the identification of the inner malformation types precisely.

## Introduction

Inner ear malformation (IEM) is a niche topic within cochlear implantation. The incidence of IEM among the sensorineural hearing loss (SNHL) population has been reported as 20–30%^1^. Cochlear implant (CI) use has been shown to restore hearing in subjects with IEM with a functional auditory nerve^2^.

Identification of IEM types is a complex process that requires the compilation of a series of computer tomography (CT) or magnetic resonance imaging (MRI) image slices to create a three-dimensional (3D) representation of the anatomical structures. It is a mentally challenging task to compile this series; to bring the sequence of the images to mind, to understand the anatomical structures, and to identify the IEM types. Another issue is that every IEM type is associated with certain surgical or CI electrode insertion complications, which makes knowing the type of IEM essential prior to CI surgery^3^.

3D segmentation of the complete inner ear structures from the clinical radiographs is one way of getting a better visualization of the anatomical structures of the IEM types, as reported by Dhanasingh et al. (2019)^4^. However, specific information on identifying the individual IEM types from the 3D images was not reported. Measuring the cochlear basal-turn diameter (A-value) in the ‘cochlear view’ in assessing the cochlear size has been reported by numerous clinics worldwide and was summed-up recently by Khurayzi et al.^5^. In that report, the A-value measurement was similar in many of the IEM types other than cochlear hypoplasia (CH), with little difference to the normal anatomy (NA) cochleae^5^. Liu et al. reported that in CH type malformation, the A-value is significantly smaller than the NA^6^. These two reports on the A-value measurement in IEM types show clearly that A-value alone is not an accurate predictor of the IEM types. Consequently, we can see that there is a need in clinical practice for a simple and effective method to identify IEM types from pre-operative CT/MRI images.

We hypothesized that just like fingerprints that are unique to every human being, each IEM type has unique landmarks/patterns that identify it as a particular type of IEM. Thus, we captured the outer contour of the inner ear in various anatomical types (normal and IEM) in the mid-modiolar section in the axial plane of CT images. This view is perpendicular to the oblique coronal plane/cochlear view in which the A-value of the cochlea can be measured. The outer contours of the inner ear were compared to common objects to facilitate the ease of identification of the IEM types and an atlas database of the outer contours of the inner ears of various anatomical types was subsequently created.

## Materials and methods

### Image analyses

Pre-operative CT scans of temporal bones of seventy-eight ears of CI candidates identified with various inner ear anatomical types were taken from the image database of King Abdullah Ear Specialist Center, King Saud University. This study was approved by the institutional review board (No. 20/0091/IRB) on 30-11-2020, to use the images after anonymization. All the CT images used were collected from 2016 to 2021. The CT images were analyzed using 3D slicer, version 4.10.2, freeware (https://www.slicer.org/). Ten samples from each of the anatomical types including, normal anatomy (NA), enlarged vestibular aqueduct syndrome (EVAS), incomplete partition (IP) type I, II and III, cochlear hypoplasia (CH), and cochlear aplasia (CA) with vestibular cavity were identified from the image collection. Only eight samples of classic common cavity (CC) were identified from the image collection.

**Figure 1:**
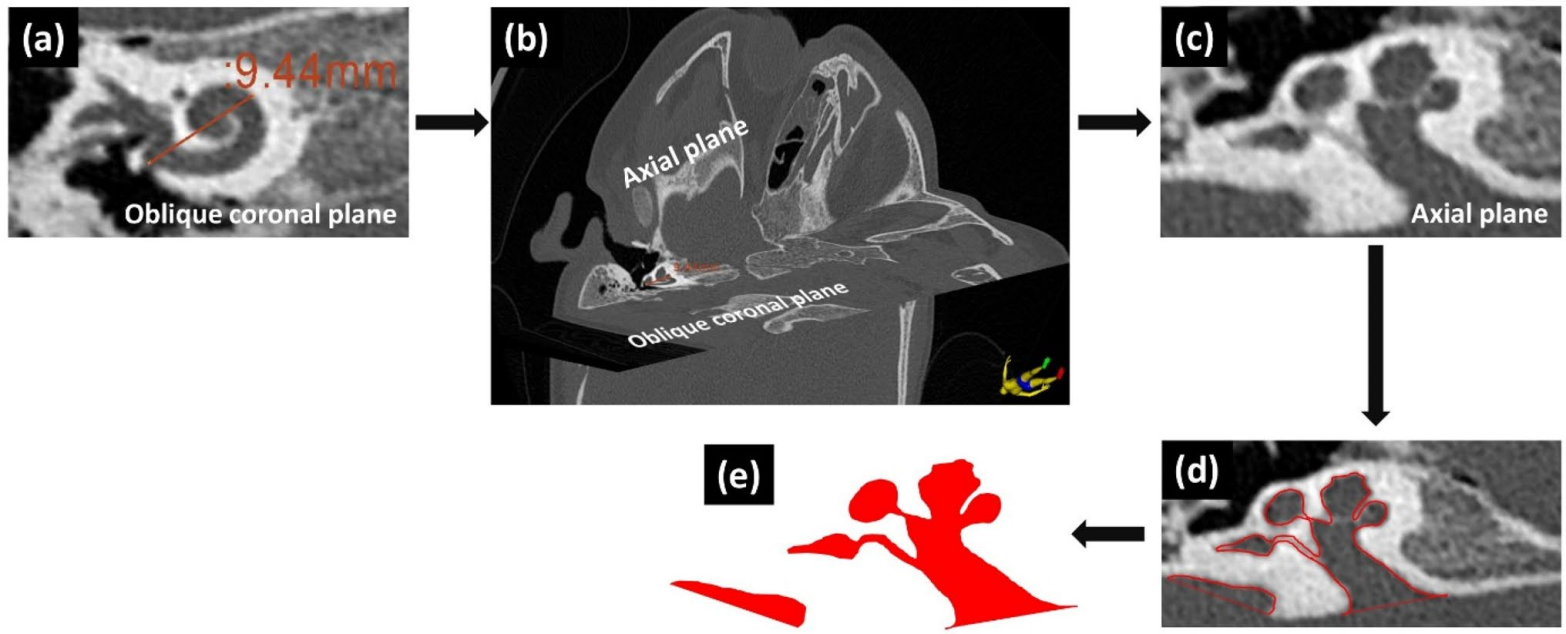
Steps involved in capturing the outer contour of the inner ear in the axial plane. Step-1: A-value measurement in the oblique coronal plane (**a**). Step-2: Along the A-value line and perpendicular to the oblique coronal plane is the axial plane (**b**). Step-3: Capture the axial plane from the step 2 (**c**). Step-4: Drawing manually along the grey-scale difference between the fluid filled and the bony region to capture the outer contour of the inner ear (**e**). Step-4: Filling the outer contour with color to finish the overall process of capturing the outer contour of the inner ear in the axial plane (**e**).

The A-value was measured in the oblique coronal plane starting at the entrance of the round window opening and passing through the center of the cochlea to the opposite side of the lateral wall in ‘cochlear view’ as described by Escude et al.^7^ and shown in Fig. 1a. Along the A-value line and perpendicular (90°) to the oblique coronal plane, the mid-modiolar section of the inner ear was visualized in the axial plane as shown in Fig. 1b. The mid-modiolar section of the inner ear captured in the axial view is shown separately in Fig. 1c. The outer contour of the mid-modiolar section of the inner ear in the axial view was captured manually by following the contrasting grey area between the fluid filled and bony region as shown in Fig. 1d. The outer contour is shown separately in Fig. 1e. This procedure was applied to all seventy-eight inner ear CT datasets to capture their outer contour.

## Results

### Demographics

Table 1 lists the various anatomical types identified, number of ears analyzed per anatomical type, mean A-values and its range for other than classic CC and CA anatomical types. Age and gender was not made available due to data protection policies of the clinic.

**Table 1:** Anatomical types of the inner ear identified along with the A-values.

Anatomical types and no. of CT scans	Mean A-value ± std (mm)	Range A-value (mm)
NA (10)	9.01 ± 0.53	8.05–9.72
EVAS (10)	9.22 ± 0.35	8.73–9.63
IP type II (10)	8.66 ± 0.59	7.78–9.48
IP type I (10)	8.32 ± 0.48	7.77–9.33
IP type III (10)	8.10 ± 0.53	7.16–8.96
CH (10)	7.38 ± 0.94	5.46–8.27
Classic CC (8)	n/a	n/a
CA (10)	n/a	n/a

*NA* normal anatomy; *EVAS* enlarged vestibular aqueduct syndrome; *IP* incomplete partition; *CH* cochlear hypoplasia; *CC* common cavity; *CA* cochlear aplasia; *n/a* not applicable.

### Outer contour of the inner ear

**Figure 2:**
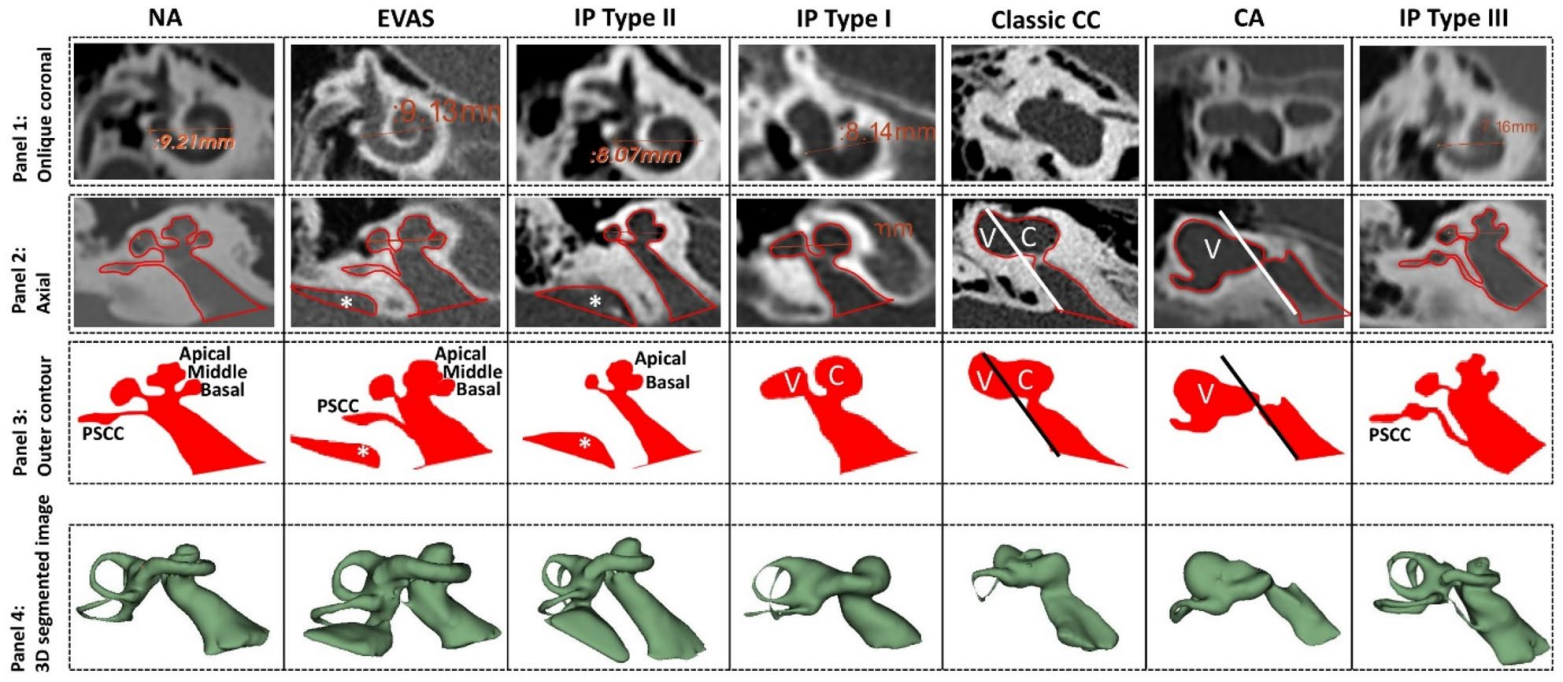
Sample data from every anatomical type in the oblique coronal view showing the A-values is shown in panel 1. Axial view showing the outer contour of the inner ear drawn manually (panel 2) and the captured outer contour filled with color (panel 3). The A-value is not measurable in the classic common cavity (CC) and vestibular cavity anatomical types. The straight line drawn parallel to the posterior edge of internal auditory canal differentiates the cochlear (C) from the vestibular portion (V) in classic CC and shows only the presence of V in the CA anatomical type. The asterisk (*) refers to the enlarged vestibular aqueduct seen in enlarged vestibular aqueduct syndrome (EVAS) and the incomplete partition (IP) type II. The 3D segmented image of the inner ear allows, performed following the procedure of Dhanasingh et al.^4^, gives a better understanding of how the inner ear structures look (panel 4). The 3D segmentation images are not to scale.

The inner ears of the various anatomical types in the oblique coronal plane/cochlear view and the A-value measurements are shown in Fig. 2 (panel 1). The corresponding axial view from which the outer contour of the mid-modiolar section of the inner ear was captured manually is shown in Fig. 2 (panel 2). The captured outer contour filled with color is shown in Fig. 2 (panel 3) and the 3D segmented image of the inner ear is shown in Fig. 2 (panel 4). The outer contour of each of the inner ear anatomical types captured from the axial plane shows a specific pattern which is explained as follows in greater detail respective to each anatomical type:

#### Normal anatomy

**Figure 3:**
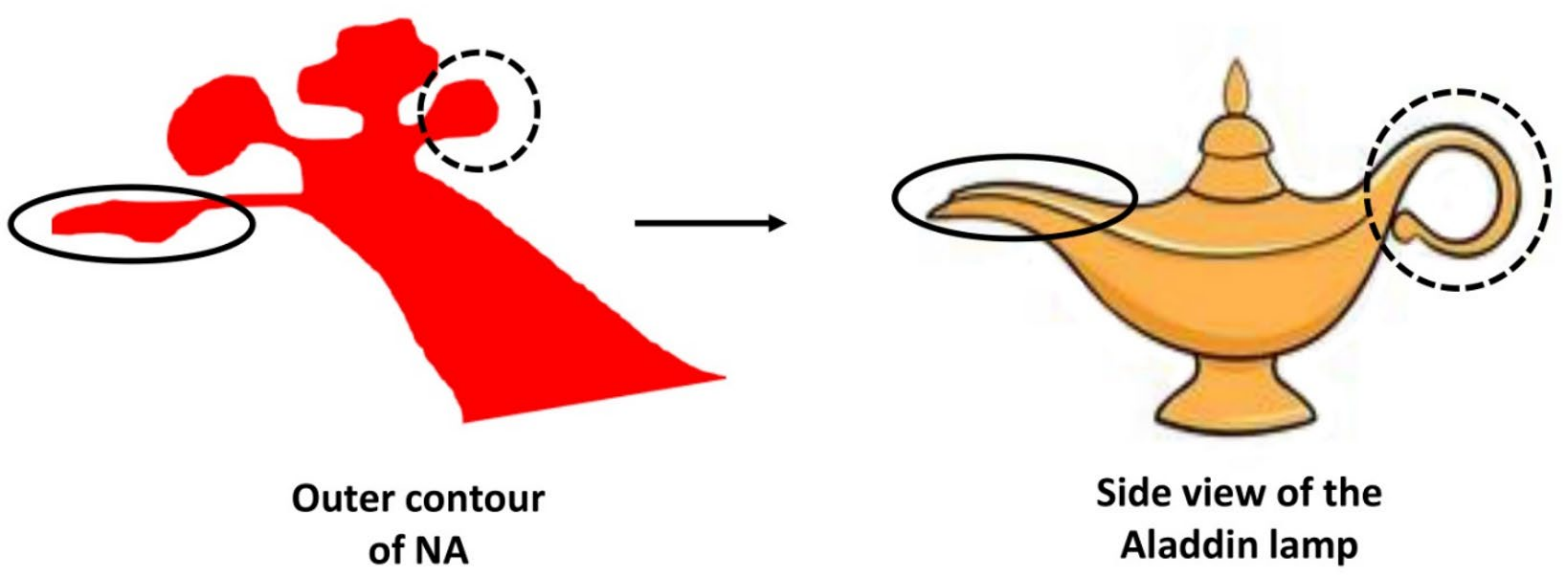
Mid-modiolar section of the cochlear portion of the normal anatomy (NA) inner ear resembles the side view of Aladdin lamp. Images not to scale.

The NA inner ear is characterized by the distinct presence of a basal turn along with the posterior semicircular canal (PSCC). Beyond the basal turn, some distinction between the middle and the apical turn can be seen. The vestibular aqueduct (VA) is usually not seen clearly in the axial plane due of its smaller width. The outer contour of the cochlear portion seen in the axial view resembles the side view of Aladdin’s lamp (Fig. 3). The PSCC can be seen as the spout/opening of the lamp, while the basal turn at 180° of angular insertion depth can be visualized as the handle.

#### Enlarged vestibular aqueduct syndrome

**Figure 4:**
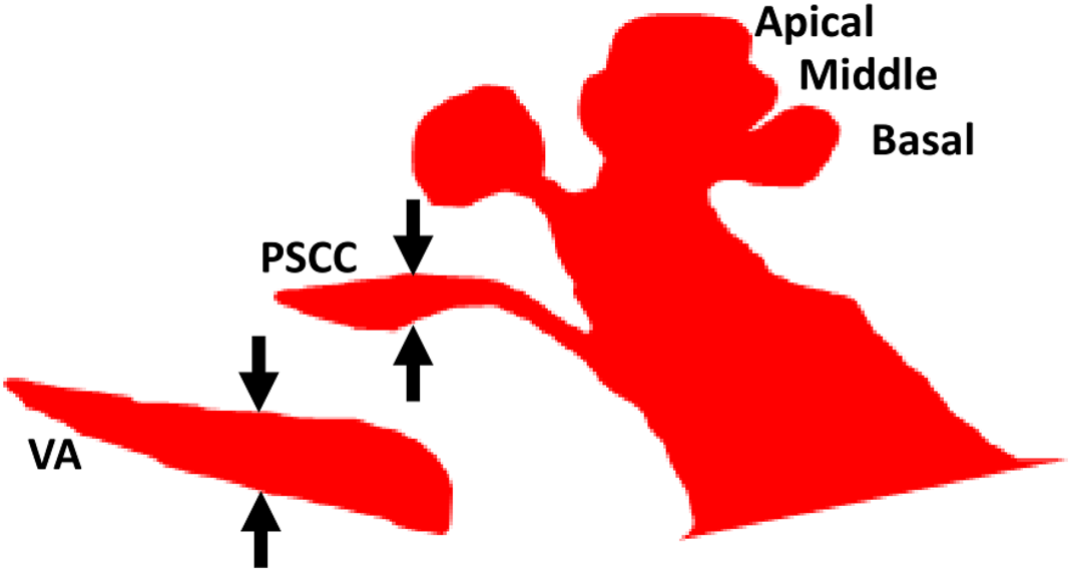
Mid-modiolar section of enlarged vestibular aqueduct syndrome (EVAS) showing the vestibular aqueduct (VA) wider than the width of the posterior semicircular canal (PSCC).

In EVAS, the basal turn is seen clearly along with some distinction between the middle and the apical turn, and the PSCC can be seen clearly in the outer contour, captured from the mid-modiolar section. It also resembles Aladdin’s lamp as shown in Fig. 4. However, the VA is wider than the width of the PSCC captured prominently in the axial view, as depicted by the black arrows shown in Fig. 4.

#### Incomplete partition type II

**Figure 5:**
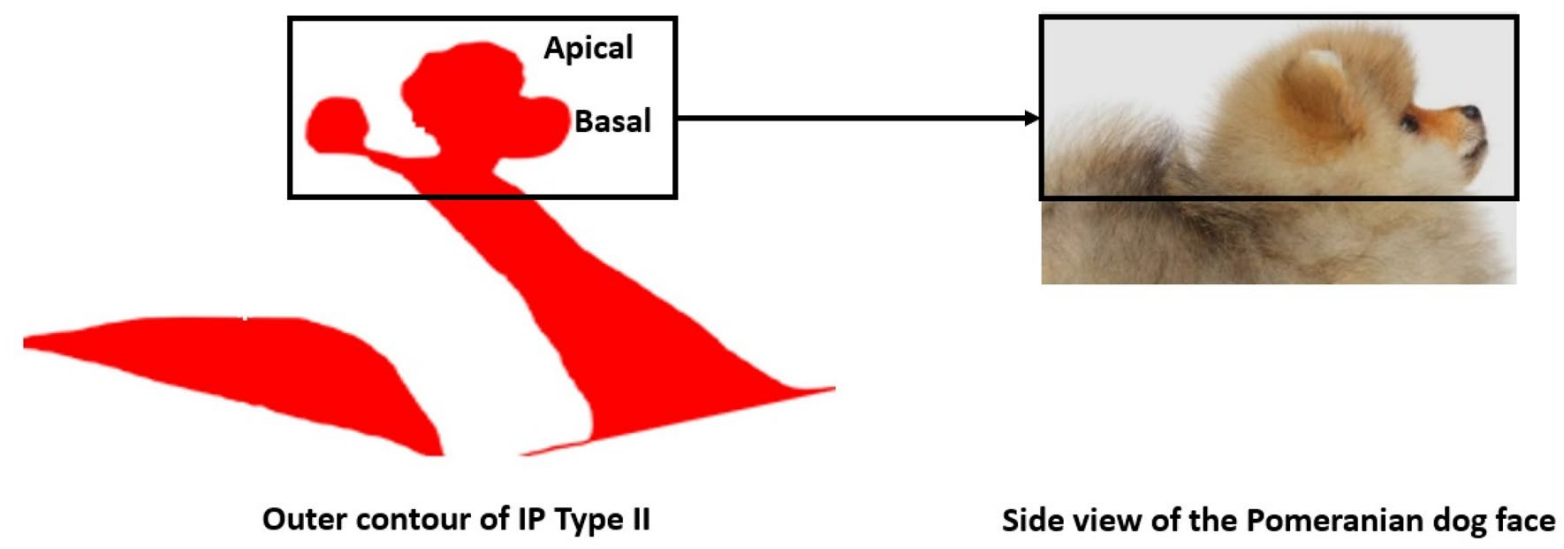
Mid-modiolar section of the cochlear portion of incomplete partition (IP) type II resembles the side view of a Pomeranian dog’s face. Images are not to scale.

In IP type II, the middle and apical turn are merged into a single cystic cavity. The outer contour of the cochlear portion, captured in the mid-modiolar section only, resembles the side view of a Pomeranian dog’s face. This is a unique feature of the IP type-II malformation as shown in Fig. 5. The enlarged VA always accompanies this malformation type.

#### Incomplete partition type I

**Figure 6:**
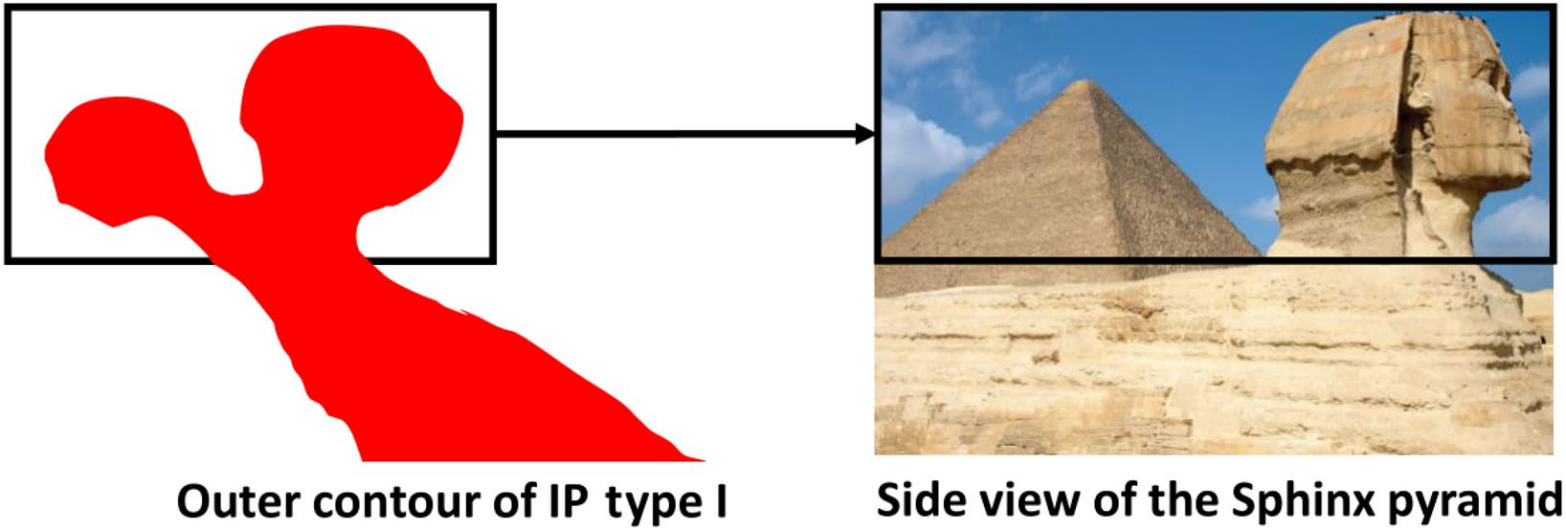
Mid-modiolar section of incomplete partition (IP) type I resembles the side view of the Sphinx pyramid. Images are not to scale.

In IP type I, in the mid-modiolar section, the cochlear portion and the vestibule are clearly separated, and both are cystic. The outer contour resembles the side view of the Sphinx pyramid. This is a unique feature of IP type I as shown in Fig. 6.

#### Classic common cavity

In the axial view, the straight-line drawn parallel to the posterior margin of the internal auditory canal (IAC) in the outer contour bisects the cavity into the cochlear and the vestibular portion; this is a unique feature of this type of malformation type as shown in Fig. 2 (5^th^ column).

#### Cochlear aplasia

In cases of CA, in the axial view, the cavity positions more posteriorly and falls under the straight-line drawn parallel to the posterior margin of the IAC; this is a unique feature of the CA malformation type as shown in Fig. 2 (6^th^ column).

#### Incomplete partition type III

**Figure 7:**
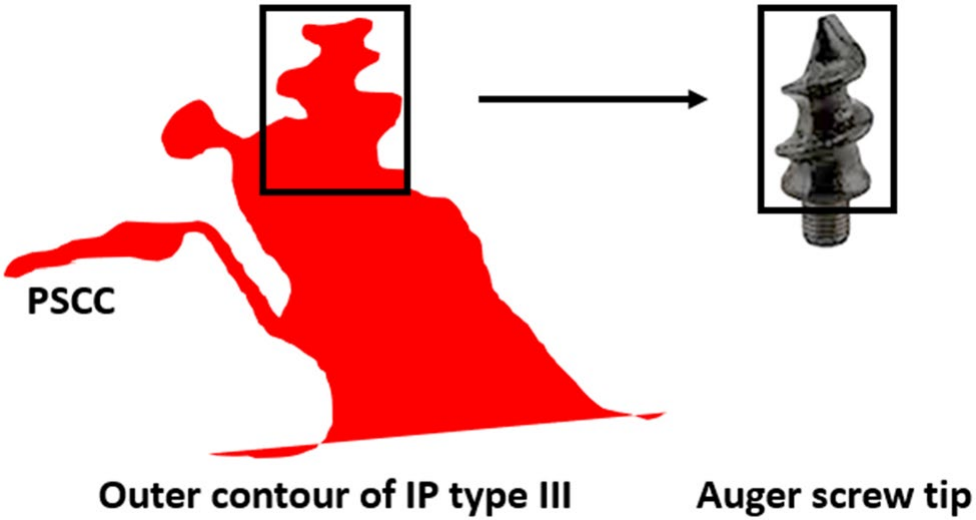
Mid-modiolar section of the cochlear portion of incomplete partition (IP) type III resembles an Auger screw tip. The width of the inner auditory canal (IAC) is as wide as the cochlear basal turn. Images are not to scale.

IP type III is the result of a genetic disorder and is characterized by steep spiraling of the cochlear turns beyond the basal portion and by a wider than normal connection between the IAC and basal turn of the cochlea. The cochlear portion in the outer contour captured in the mid-modiolar section appears like an Auger screw tip and this is the unique feature of this malformation type as shown in Fig. 7. The PSCC can usually be visualized along with the inferior vestibular nerve branch from the IAC connecting it.

#### Cochlear hyperplasia

**Figure 8:**
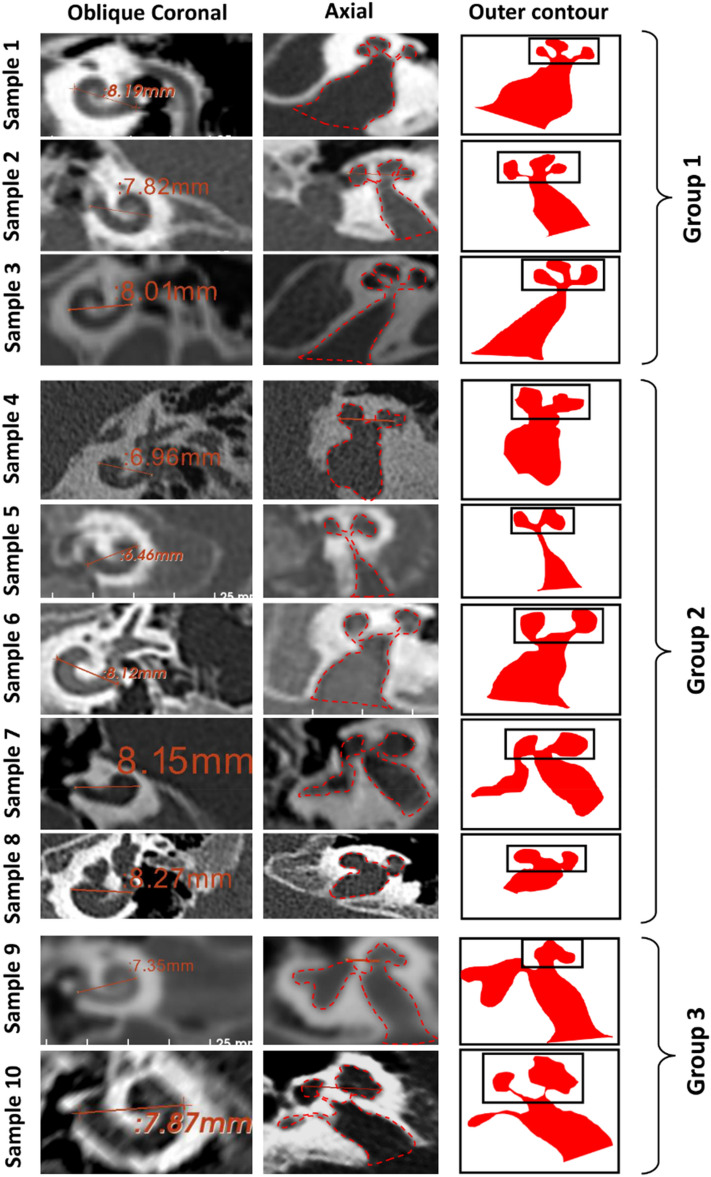
Cochlear hypoplasia (CH) type malformation shows a huge variation in the size, shape, and the availability of cochlear and vestibular portion. The outer contour of the mid-modiolar sections of the ten samples were brought under three groups. The outer contour of group 1 resembles a maple leaf, group 2 resembles a distorted dumbbell and the group 3 shows no close resemblance to any familiar shape.

Within the samples made available under this malformation type, a huge variation in the size, shape, and the availability of the cochlear and vestibular portions was identified. There was no single unique feature seen in the outer contour of the mid-modiolar section common to all samples (Fig. 8). The outer contour of samples 1–3 showed a better development of the cochlear portion beyond the basal turn resembling a maple leaf and were placed under group 1. Samples 1–3 lacked the vestibular portion completely. Samples 4–8 showed minimal development of the basal turn of the cochlea, and the outer contour of the mid-modiolar section of the cochlea in the axial view somewhat resembled a distorted dumbbell. Samples 9 and 10 show prominent development of the cochlear portion and it was difficult to find an object that resembled it. Sample 10 showed stenosis in the fundus region.

### Image database/atlas

**Figure 9:**
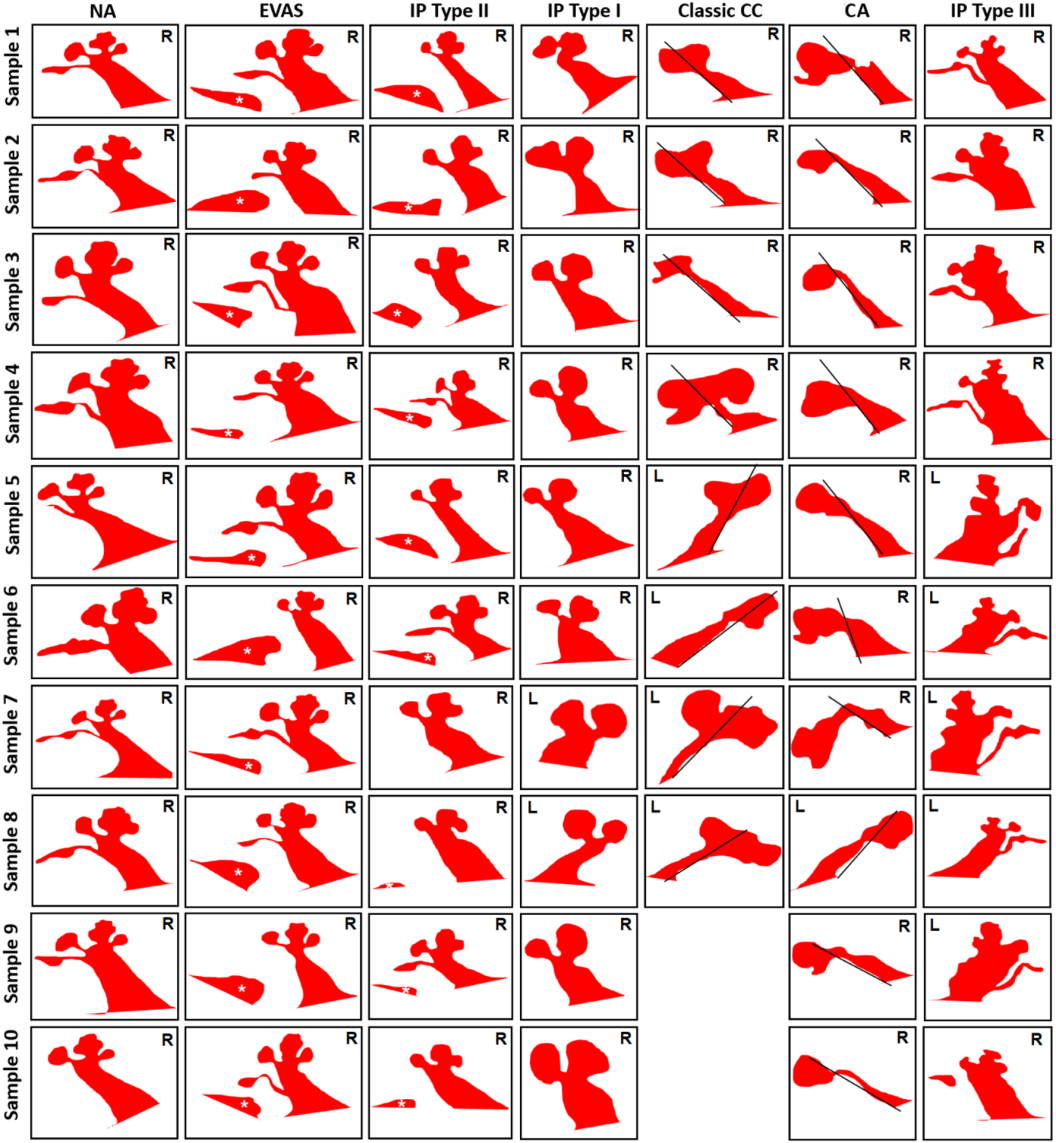
Atlas of outer contours of mid-modiolar section of inner ear of different anatomical types. Classic CC has eight samples whereas other inner ear malformation types have ten samples. White asterisk refers to the vestibular aqueduct and the black line separates the cochlear and the vestibular portion in classic common cavity (CC) and shows the presence of vestibular cavity in cochlear aplasia (CA) type malformations. The white asterisk points to the enlarged VA. Normal anatomy: NA, Enlarged vestibular aqueduct syndrome: EVAS, incomplete partition: (IP) type I, II and III, Cochlear hypoplasia: CH. Images are not to scale. R: right ear; L: left ear.

Figure 9 is a collection of the outer contours of the mid-modiolar sections of various IEM types other than CH reported in this study. CC had only eight samples, whereas all the other anatomical types comprised ten samples each. The unique signature identified with each of the anatomical types is shown in this atlas of images.

## Discussion

We present herein a novel method of identifying IEM types from the outer contour of the mid-modiolar section of inner ear viewed in the axial plane, corresponding to the oblique coronal plane in which the A-value is measured. Interestingly, the outer contour of the mid-modiolar section of various anatomical types somewhat resembles a common object with which the easy identification of the IEM types can be facilitated.

In the NA cochlea, the mid-modiolar section of the cochlea could be related to the side view of Aladdin’s lamp. In the EVAS samples, the mid-modiolar section of EVAS types in the axial view looked identical to the NA in every respect, but it includes the VA in an enlarged format. It is the enlarged VA that distinguishes the NA and the EVAS types from each other.

The side view of the Pomeranian dog’s face along with an enlarged VA was used as a visual reminder of the distinguishing features of the IP type II malformations in the mid-modiolar section. This could be observed clearly in all samples, except sample 7, in which the PSCC was not observed. Whereas IP type I in its mid-modiolar section, captured from the axial view, showed the cochlear portion and the vestibule separated, resembling the profile of a Sphinx pyramid. The size of the vestibule and cochlear portion were highly variable as determined qualitatively from all ten samples. IP type III is the result of genetic disorder that is marked by the absence of the bony modiolus and the partial presence of bony interscalar septa inside the cochlear portion^8^. This, along with steep spiraling of cochlear turns gives an Auger screw tip appearance to IP type III in the mid-modiolar section. The PSCC was also visible in these samples.

In our collection, the CH type malformation showed the most variation. This finding is in-line with Halawani et al. who reported a huge variation in the size, shape, and the availability of anatomical structures with CH type malformations using 3D segmented images^9^. Liu et al. reported on the classification of CH types based on the cochlear turns available and by measuring the A-value and cochlear height in the axial plane without defining the mid-modiolar section^6^. Recently, Pamuk et al.^10^ and Khurayzi et al.^5^ reported on the shorter A-value associated with CH type compared to the NA cochlea. We were able to bring the ten samples of CH under three groups based on the outer contour of the inner ear captured in the mid-modiolar section. Not finding a single unique outer contour that fits to any CH type malformation type can be considered as a weakness of this classification, but this is mainly due to the huge anatomical variations associated with this malformation type.

Orienting the CT images to obtain the mid-modiolar section can be tricky with the classic CC and CA types, where the A-value cannot be measured in the cochlear view. This is due to the fact that these two malformation types lack the normal development of the cochlear portion as a result of embryological developmental arrests at 4^th^ week of gestation and fails to develop into the inner ear resulting in a cavity^11^. Therefore, we recommend using the standard axial view provided by any DICOM viewers in which both sides of the inner ear are visualized somewhat symmetrically. As described by Kontorinis et al.^12^ and Weiss et al.^13^, drawing a straight line parallel to the posterior margin of the IAC in the axial view would differentiate classic CC from CA. The straight line will dissect the cavity showing the cochlear and the vestibular portion clearly in the classic CC, whereas the whole cavity would fall under the straight line in the CA. This is a simple way of differentiating classic CC from CA. In our collection we found only eight samples under classic CC compared to ten samples under CA (Fig. 9).

One of the key reasons to know the IEM types precisely before CI surgery is to prepare for the intra- and post-operative complications associated with each IEM type. It would also be helpful in establishing the prognosis for hearing benefit^14^. In the ten samples provided for each IEM types, other than for classic CC of which we had eight, the outer contours were found to be distinctive for each IEM type.

For some reasons, if the true mid-modiolar section is not captured correctly, the chances are there for not seeing the outer contour resembling specific objects corresponding to every IEM types as reported in this study. Please find in supplement figure A–E corresponding to NA, EVAS, IP type I, type II, and type III anatomical types respectively, to see how the outer contour changes when the mid-modiolar section off-sets by two slices either superiorly or inferiorly. Superiorly off from the mid-modiolar section did not diminish the outer contours of known objects in NA, IP type I, type II, and type III. Whereas in EVAS, superiorly off from the mid-modiolar section diminished the outer contour to look like Aladdin lamp. Inferiorly off from mid-modiolar section did diminish the outer contours in all anatomical types other than IP type III. This emphasizes the importance of sticking to the true mid-modiolar section as described in this study to capture the outer contours precisely. The A-values for all the anatomical types reported in Table 1 has no direct relevance to the key findings of this study but the measurement process of A-values helped in obtaining the true mid-modiolar section.

## Conclusion

Our finding of capturing the outer contour of the mid-modiolar section of the inner ear from the axial view is a novel way of identifying IEM types. This requires one additional step of getting the axial view to capture the mid-modiolar section of the inner ear, to visualizing the cochlea in the ‘cochlear view’/oblique coronal plane while measuring the A-value. Each IEM type other than CH type was identified as having a unique pattern or outer contour of the mid-modiolar sections with which the IEM types could be identified.

## Supplementary Information

Supplementary Information 1.

Supplementary Information 2.

Supplementary Information 3.

Supplementary Information 4.

Supplementary Information 5.

Supplementary Information 6.
