# Supplementary figures and images for: A novel method of identifying inner ear malformation types by pattern recognition in the mid modiolar section
Source: Sci Rep. 2021 Oct 21;11:20868. doi: 10.1038/s41598-021-00330-6 (PMC8531302; doi:10.1038/s41598-021-00330-6)

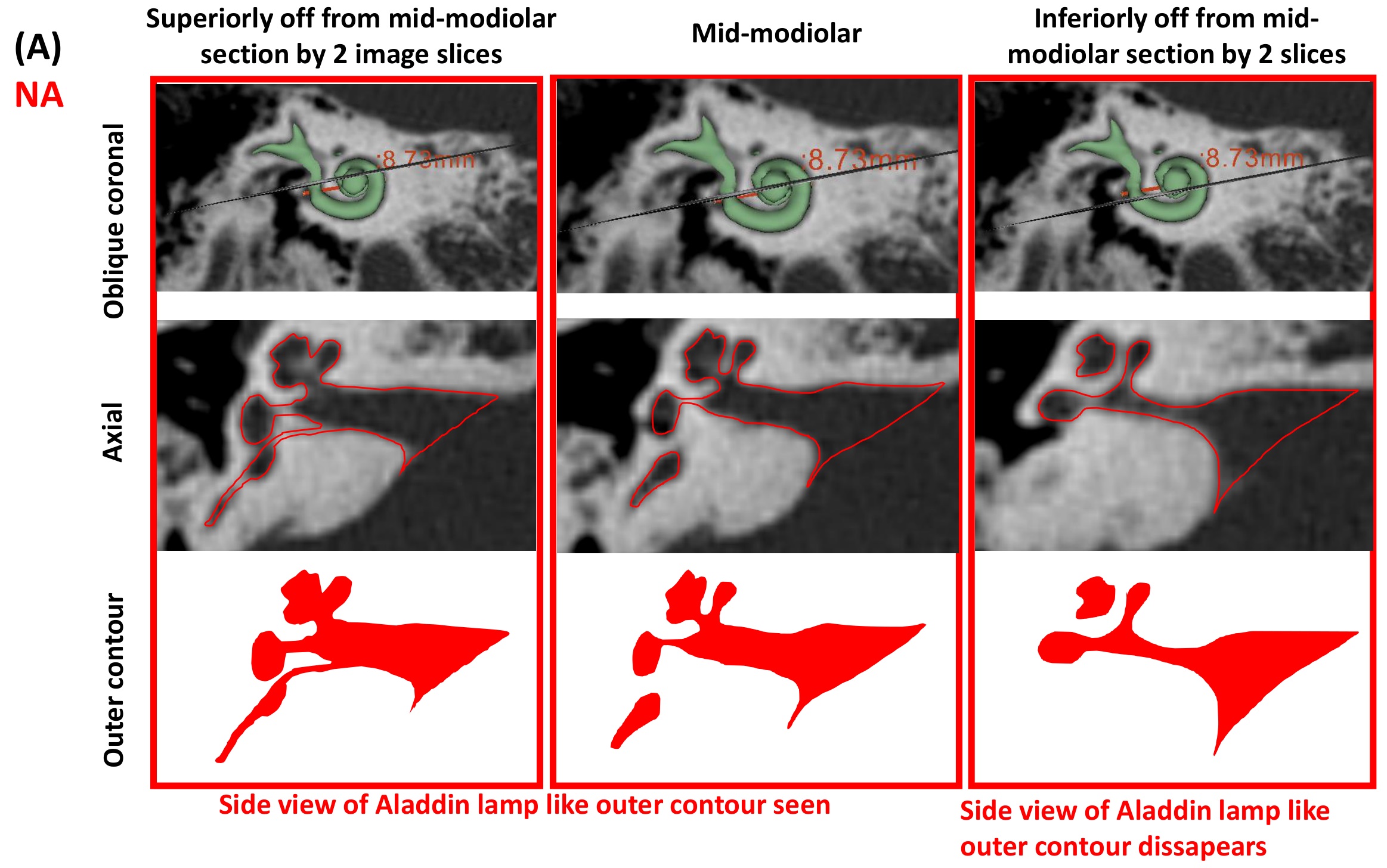

Supplement: Supplementary file 1 — Supplementary Information 1. [file 41598_2021_330_MOESM1_ESM.jpg]

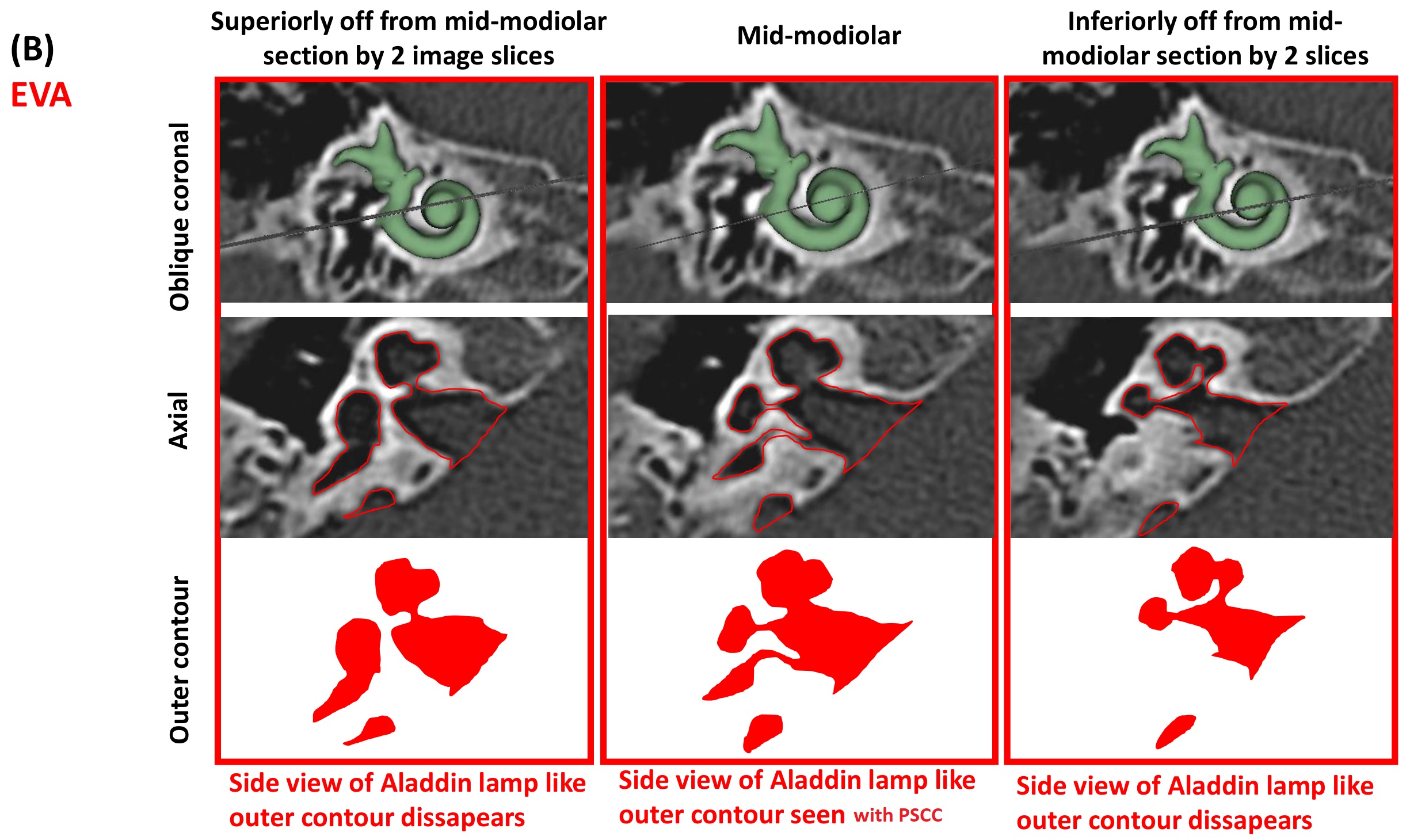

Supplement: Supplementary file 2 — Supplementary Information 2. [file 41598_2021_330_MOESM2_ESM.jpg]

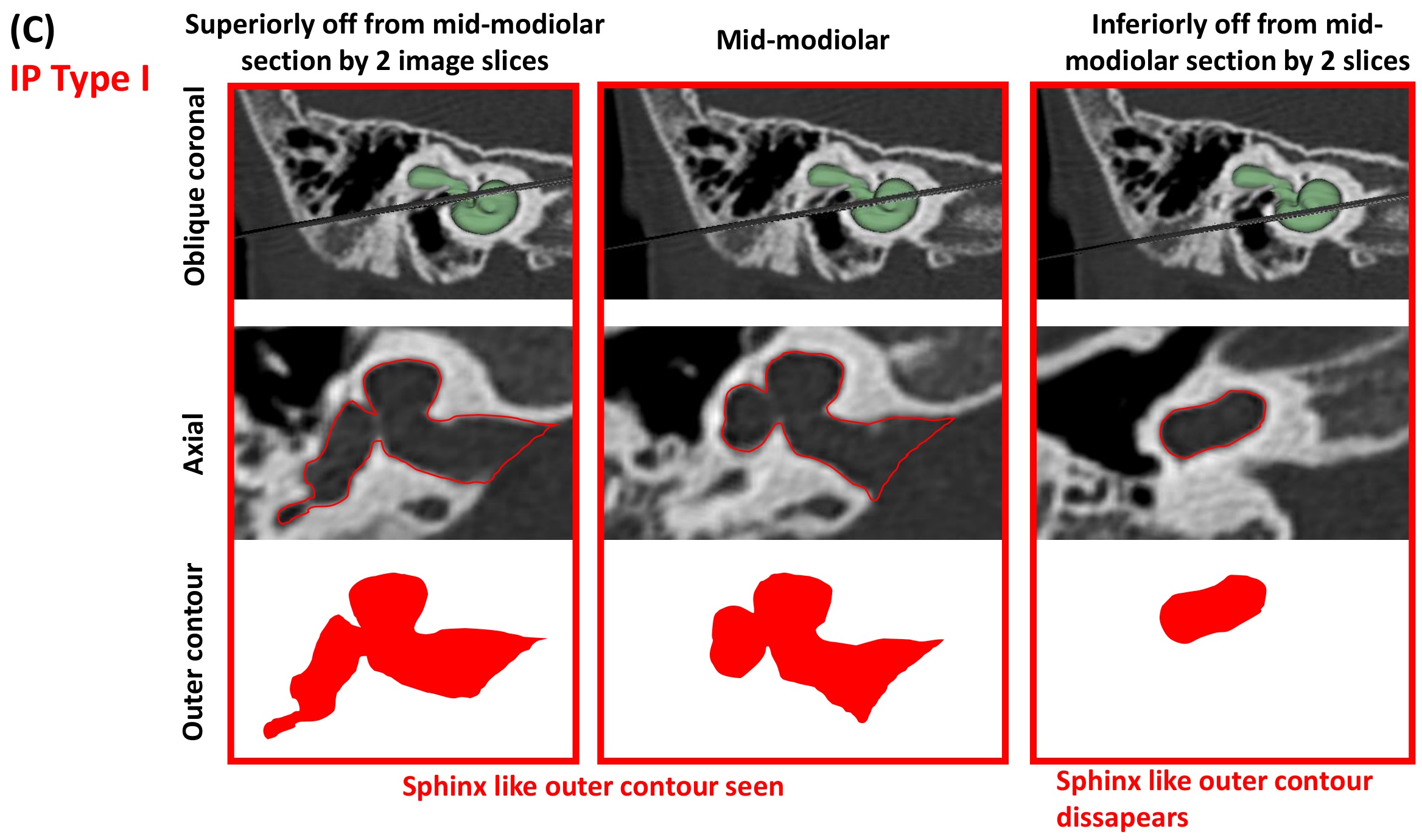

Supplement: Supplementary file 3 — Supplementary Information 3. [file 41598_2021_330_MOESM3_ESM.jpg]

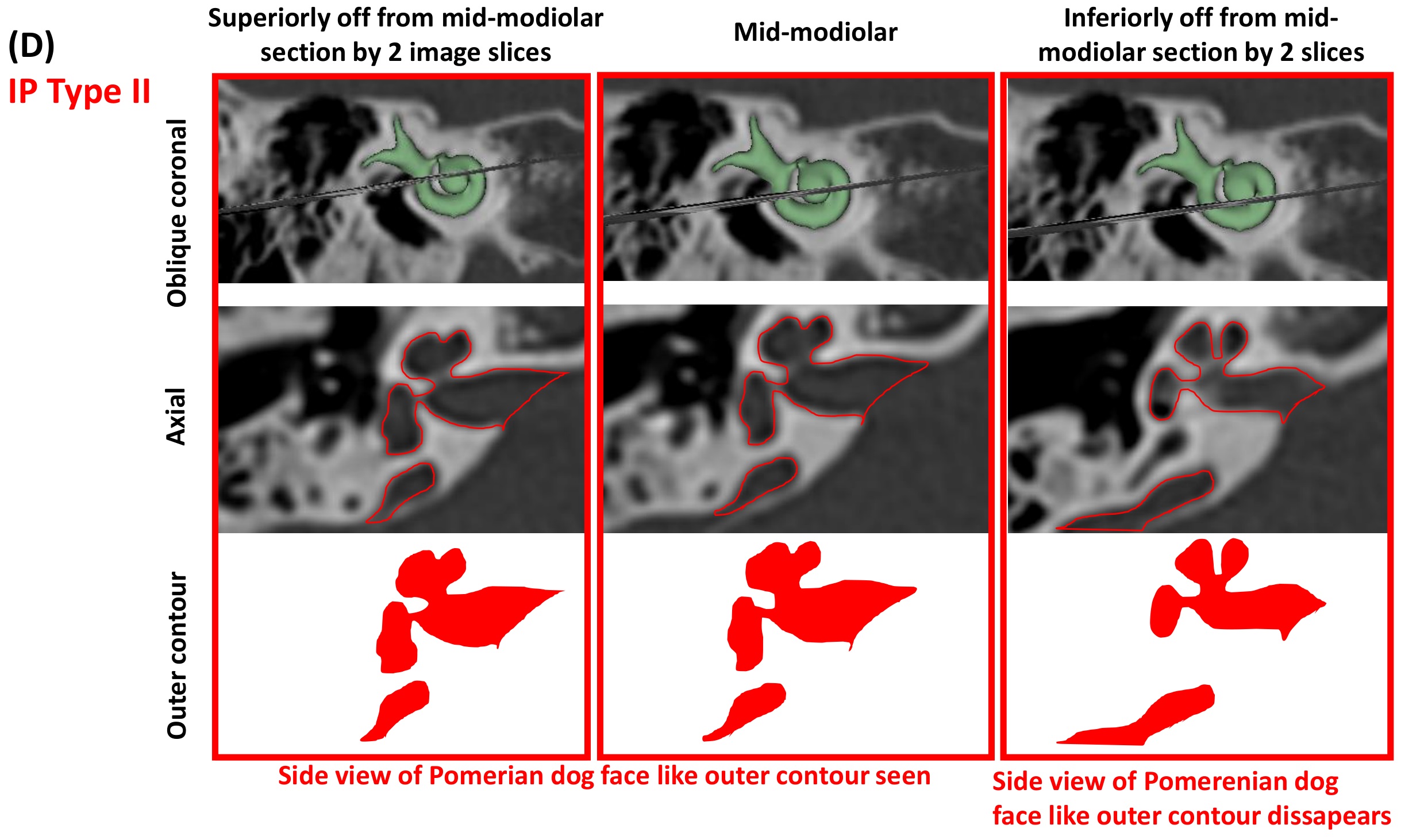

Supplement: Supplementary file 4 — Supplementary Information 4. [file 41598_2021_330_MOESM4_ESM.jpg]

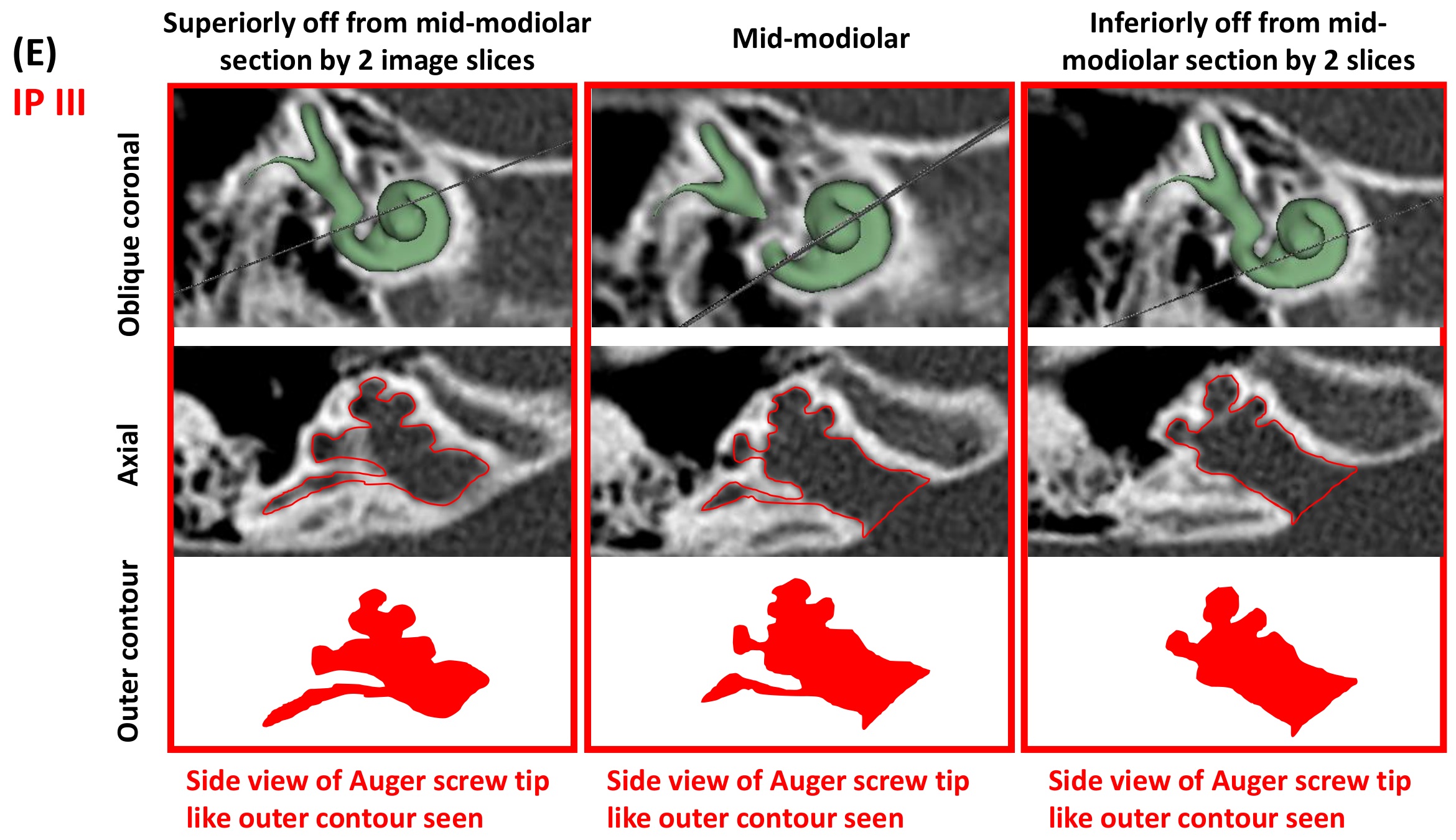

Supplement: Supplementary file 5 — Supplementary Information 5. [file 41598_2021_330_MOESM5_ESM.jpg]
