# Supplementary material for: A novel method of identifying inner ear malformation types by pattern recognition in the mid modiolar section
Source: Sci Rep. 2021 Oct 21;11:20868. doi: 10.1038/s41598-021-00330-6 (PMC8531302; doi:10.1038/s41598-021-00330-6)
Supplement: Supplementary file 6 — Supplementary Information 6. [file 41598_2021_330_MOESM6_ESM.docx]

**Supplement figure legend**

Observing the outer contours of the mid-modiolar section and the offset from the mid-modiolar section, both superiorly and inferiorly by 2 image slices of NA (A), EVAS (B), IP type I (C), IP type II (D), and IP type III (E).
